# Supplementary material for: Pervasive associations between dark septate endophytic fungi with tree root and soil microbiomes across Europe
Source: Nat Commun. 2024 Jan 2;15:159. doi: 10.1038/s41467-023-44172-4 (PMC10761831; doi:10.1038/s41467-023-44172-4)
Supplement: Supplementary file 1 — Supplementary Information [file 41467_2023_44172_MOESM1_ESM.pdf]

Supplementary Materials for

Pervasive associations of dark septate endophytic fungi with tree root and soil microbiomes across Europe

Tarquin Netherway*, Jan Bengtsson, Franz Buegger, Joachim Fritscher, Jane Oja, Karin Pritsch, Falk Hildebrand, Eveline J. Krab, Mohammad Bahram

*Corresponding author: tarquin.netherway@slu.se

This PDF file includes:

Supplementary Notes 1 to 5

Supplementary discussion

Supplementary Figs. S1 to S10

Supplementary references

Supplementary Note 1

Root symbiont colonization and their relative abundances in soil and roots

The additional results presented in Fig. S1 were a negative relationship between ectomycorrhizal (EcM) colonization and climatic moisture deficit (CMD) (R^2m = 0.23, p = 7e-05), a positive correlation between the relative abundance of EcM fungi in roots and soil moisture (R^2m = 0.22, p = 2e-04), soil C/N (R^2m = 0.32, p = 2e-04), and the coniferous EcM tree basal area (R^2m = 0.11, p = 0.025). Furthermore, there was a negative correlation between the relative abundance of EcM fungi in roots with mean annual temperature (MAT) (R^2m = 0.30, p = 5e-04) (Fig. S1), and the relative abundances of EcM fungi in soil and roots were positively correlated (R^2m = 0.39, p = 2e-05), and were associated with a similar set of biotic and abiotic parameters (Fig. S1). All three measures of EcM fungal prevalence (colonization and soil and root relative abundances) were strongly associated with moisture availability.

For arbuscular mycorrhizal (AM) fungi, the relative abundance of AM fungi in soil was positively correlated the proportion of AM fungi in roots (R^2m = 0.22, p = 0.001), AM colonization (R^2m = 0.13, p = 0.010), and negatively correlated with the basal area of EcM/AM tees (R^2m = 0.31, p = 3e-05) (Fig. S1). Despite our choice of primers not being ideal for the detection of AM fungi, all three measures of AM fungal prevalence (colonization and soil and root relative abundances) we performed were aligned in terms of correlating with one another and a similar set of biotic and abiotic variables (Fig. S1).

For dark septate endophytic (DSE) fungi, DSE colonization was additionally negatively correlated with MAT (R^2m = 0.11, p = 0.023), and was positively correlated with the basal area of coniferous EcM trees (R^2m = 0.09, p = 0.022) (Fig. S1). The relative abundance of

potential DSE fungi in roots was also positively correlated with soil C/N ($R^2m = 0.25$, $p = 0.002$) and was positively correlated with the basal area of coniferous EcM trees ($R^2m = 0.10$, $p = 0.033$) (Fig. S1).

Supplementary Note 2

The effect of tree symbioses on the relative abundance of different CAZymes

All three types of symbiont colonization appeared to have associations with the relative abundance of specific classes of CAZyme genes in soil, and only DSE colonization in roots (Fig. S5). There were negative relationships observed between EcM colonization and bacterial CAZymes targeting cellulose in soil ($R^2m = 0.22$, $p = 6e\text{-}04$), AM colonization and those targeting glucans in soil ($R^2m = 0.25$, $p = 5e\text{-}04$), DSE colonization and those targeting lignin ($R^2m = 0.25$, $p = 5e\text{-}04$), and a positive relationship between DSE colonization and bacterial CAZymes targetting glucans in soil ($R^2m = 0.25$, $p = 5e\text{-}04$) (Fig. S5). In roots there were negative relationships observed between DSE colonization and the relative abundance of bacterial CAZymes targeting cellulose ($R^2m = 0.24$, $p = 0.001$), hemicellulose ($R^2m = 0.34$, $p = 9e\text{-}05$), lignin ($R^2m = 0.30$, $p = 2e\text{-}04$), chitin ($R^2m = 0.26$, $p = 7e\text{-}04$), glucans ($R^2m = 0.27$, $p = 0.001$), and peptidoglycans ($R^2m = 0.42$, $p = 8e\text{-}05$) (Fig. S5).

Supplementary Note 3

The effect of tree symbioses on the relative abundance of N cycling genes

When looking at the relative abundance of different groups of N cycling genes involved in different processes (organic N degradation, assimilatory nitrate reduction, dissimilatory nitrate reduction, denitrification, nitrification, N fixation, organic N synthesis, and hydroxylamine reduction), DSE colonization appeared to be one of the stronger and more consistent factors influencing N cycling genes in soil, and an even more pronounced effect in

roots (Fig. S6), while AM and EcM colonization had very few significant correlations with N cycling genes (Fig. S6). In soil DSE colonization had a negative correlation with the proportion of organic N degradation ($R^2m = 0.20$, $p = 0.002$), assimilatory nitrate reduction ($R^2m = 0.27$, $p = 8e\text{-}05$), dissimilatory nitrate reduction ($R^2m = 0.32$, $p = 1e\text{-}04$), denitrification ($R^2m = 0.40$, $p = 9e\text{-}06$), nitrification ($R^2m = 0.22$, $p = 0.001$), and hydroxylamine reduction genes ($R^2m = 0.11$, $p = 0.031$), and AM colonization had a positive correlation with the proportion of organic N degradation genes in soil ($R^2m = 0.26$, $p = 1e\text{-}04$). In roots DSE also had negative correlation with organic N degradation ($R^2m = 0.36$, $p = 6e\text{-}04$), assimilatory nitrate reduction ($R^2m = 0.20$, $p = 2e\text{-}04$), dissimilatory nitrate reduction ($R^2m = 0.43$, $p = 0.002$), denitrification ($R^2m = 0.64$, $p = 1e\text{-}09$), nitrification ($R^2m = 0.51$, $p = 1e\text{-}04$), N fixation ($R^2m = 0.16$, $p = 0.014$), organic N synthesis ($R^2m = 0.33$, $p = 1e\text{-}04$), and hydroxylamine reduction genes ($R^2m = 0.40$, $p = 1e\text{-}05$).

Supplementary Note 4

The effect of tree symbioses on the proportions of P cycling genes

When considering P cycling genes involved in specific processes (organic P mineralization, inorganic P mobilization, and P starvation response regulation) in soil these genes were correlated most strongly with soil pH, and only EcM colonization had a significant (negative) correlation with inorganic P mobilization genes ($R^2m = 0.12$, $p = 0.022$) out of the root colonization types (Fig. S7). In roots, DSE colonization had a negative correlation with organic P mineralization ($R^2m = 0.36$, $p = 4e\text{-}05$), inorganic P mobilization ($R^2m = 0.21$, $p = 0.003$), and P starvation response regulation genes ($R^2m = 0.31$, $p = 3e\text{-}05$), whereas EcM and AM did not show significant associations (Fig. S7).

Supplementary Note 5

The effect of abiotic and biotic factors on tea bag decomposition

After various iterations of variable selection using random forest, Spearman rank correlation analysis, and forward selection before testing individual relationships using linear mixed-effects models, the main abiotic parameters associated with rooibos mass loss in mixed-effects models were soil $\delta^{13}C$ ($R^2m = 0.59$, p = 1e-07) and MAT ($R^2m = 0.32$, $p = 0.001$) with positive correlations, compared to soil moisture ($R^2m = 0.24$, $p = 0.001$) with a negative correlation (Fig. S8). Whereas green tea mass loss was only positively correlated with soil $\delta^{13}C$ ($R^2m = 0.21$, $p = 0.017$) and MAP ($R^2m = 0.36$, $p = 0.004$) (Fig. S8). Out of the three colonization types, DSE colonization had a positive correlation ($R^2m = 0.12$, $p = 0.026$) and EcM colonization had a negative correlation ($R^2m = 0.21$, $p = 0.010$) with rooibos mass loss (Fig. S8), while none of the colonization types had a significant correlation with green tea mass loss. When looking at the relationships between various soil and root microbiome properties with rooibos mass loss we found a negative correlation with the relative abundance of EcM fungi in both roots ($R^2m = 0.24$, $p = 0.003$) and the EcM fungal genera *Suillus* ($R^2m = 0.21$, $p = 0.006$), and *Helvellosebacina* ($R^2m = 0.15$, $p = 0.006$) in soil (Fig. S8). In comparison, positive correlations were observed with the relative abundance of the saprotrophic fungal genus *Acremonium* in soil ($R^2m = 0.16$, $p = 0.014$), the soil bacterial CAZymes GT20 ($R^2m = 0.45$, p = 1e-04), GH5_24 ($R^2m = 0.35$, p = 4e-04), PL12_3 ($R^2m = 0.19$, $p = 0.005$), and GH_6 ($R^2m = 0.32$, p = 1e-04), and the soil fungal CAZymes GH43_26 ($R^2m = 0.36$, p = 1e-04), AA3_2 ($R^2m = 0.24$, $p = 0.001$) and GH25 ($R^2m = 0.16$, $p = 0.011$). In addition, there were also positive correlations between rooibos mas loss and the root bacterial CAZymes PL5 ($R^2m = 0.24$, $p = 0.003$) and GT45 ($R^2m = 0.19$, $p = 0.003$), the root fungal CAZymes AA11 ($R^2m = 0.31$, $p = 0.003$), GH134 ($R^2m = 0.58$, p = 1e-04), GH81 ($R^2m = 0.36$, $p = 0.001$), PL20 ($R^2m = 0.17$, $p = 0.022$), GH26 ($R^2m = 0.23$, $p = 0.010$) and GH92 ($R^2m = 0.20$, $p = 0.010$) (Fig. S8). There was also a positive association for the

diversity of fungal CAZymes in roots ($R^2m = 0.21$, $p = 0.008$) (Fig. S8). For green tea mass loss, we only observed a positive correlation with the proportion of the bacterial CAZyme family GT45 ($R^2m = 0.12$, $p = 0.028$) in roots, and the fungal CAZyme family GH134 ($R^2m = 0.32$, $p =$ 1e-04) in roots (Fig. S8).

Supplementary discussion

Our findings linking EcM fungi and moisture availability are in line with a global metanalysis showing EcM colonization patterns to be associated with more constant precipitation [1] and experimental local-scale and mesocosm studies showing a reduction in EcM colonization following drought treatments [2-4]. Together, several lines of evidence suggest that the EcM symbiosis is sensitive to low-moisture levels and may be profoundly impacted by climate change. However, the EcM symbiosis may buffer the effects of drought on host plants [5], potentially through a shift to EcM fungi with more extensive extraradical mycelia compared to contact types [6, 7]. Yet it is difficult to separate the effects of EcM on water transport from nutritional effects, which become more important with decreasing soil moisture [8].

Our relative abundance measure of DSE in roots and DSE colonization were also aligned with one another, and overall, our findings on DSE associations suggests that DSE colonization is highest in harsh conditions of low-nutrient availability (high soil C/N and low pH) and harsh climates; hinting at a potential role in plant stress tolerance in natural ecosystems. They have previously been found to have higher colonization rates with increasing soil organic matter content [9], and have been suggested to be bioindicators of colder climates [10].

Supplementary figures

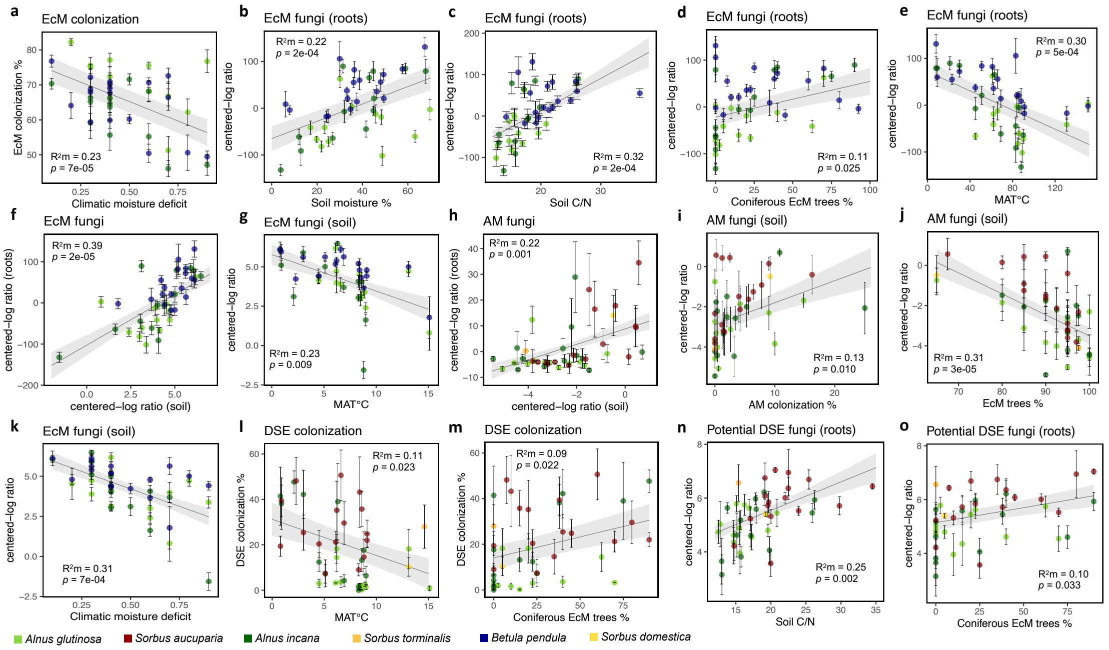

Fig. S1 Results of linear mixed-effects models showing **(a)** ectomycorrhizal (EcM) colonization (% root tips colonized) as explained by climatic moisture deficit, (*n* = 43), **(b)** the relative abundance (centered-log ratio) of EcM fungi (metabarcoding reads) on the roots as explained by soil moisture content (*n* = 43), **(c)** the relative abundance of EcM fungi on roots as explained by soil carbon/nitrogen (C/N) (*n* = 43), **(d)** the relative abundance of EcM fungi on roots as explained by the basal area of coniferous EcM trees (*n* = 43), **(e)** the relative abundance of EcM fungi on roots as explained by mean annual temperature (*n* = 43), **(f)** the relative abundance of EcM fungi on roots as explained by the relative abundance of EcM fungi in soil (*n* = 43), **(g)** the relative abundance of EcM fungi in soil as explained by mean annual temperature (*n* = 43), **(h)** the relative abundance of arbuscular mycorrhizal (AM) fungi on roots as explained by the relative abundance of AM fungi in soil (*n* = 43), **(i)** the relative abundance of AM fungi in soil as explained by AM colonization (% root length colonized) (*n* = 43), **(j)** the relative abundance of AM fungi in soil as explained by the EcM/AM tree basal area, **(k)** the relative abundance of EcM fungi in soil as explained by climatic moisture deficit (*n* = 43), **(l)** dark septate endophyte (DSE) colonization (% root length colonized) as

explained by mean annual temperature, (*n* = 43), **(m)** DSE colonization as explained by the basal area of coniferous EcM trees (*n* = 43), **(n)** the relative abundance of potential DSE fungi on roots as explained by soil C/N (*n* = 43), **(o)** the relative abundance of potential DSE fungi on roots as explained by the basal area of coniferous EcM trees (*n* = 43). Colors represent different tree hosts: *Betula pendula* (blue), *Sorbus* aucuparia (red), *S. torminalis* (orange), *S. domestica* (yellow), *Alnus glutinosa* (light green), and *A. incana* (dark green). Data points (*n*) presented are mean values of individual tree samples (> 10 m apart) from biologically independent tree species at each independent site (i.e., *n* = tree species X site), and error bars represent +/- the standard error (SE) of the mean. The marginal R^2 (R^2m) of the fixed effect and *p* values (calculated using the Satterthwaite approximation in a two-sample *t*-test) for each linear mixed-effects model (plot embedded in site crossed with tree species as random effects) are listed, and the standard error of the fitted line is shaded grey. The statistical test used was two-sided. For linear-mixed effects model summaries supporting this figure see Supplementary Data 13.

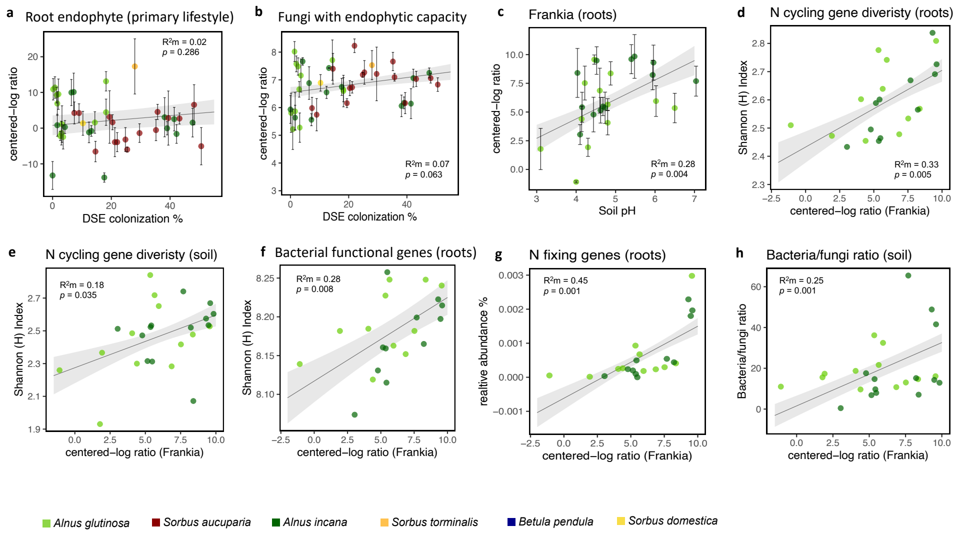

Fig. S2 Results of linear mixed-effects models showing **(a)** the relative abundance (centered-log ratio of metabarcoding reads) of fungal root endophytes (primary lifestyle) on the roots as explained by dark septate endophyte (DSE) colonization (*n* = 43), **(b)** the relative abundance of fungi with potential endophytic capacity on roots as explained by DSE colonization (*n* = 43), **(c)** the relative abundance of *Frankia* bacteria on roots as explained by soil pH (*n* = 25), **(d)** the diversity (Shannon H Index) of nitrogen (N) cycling genes on roots as explained by the relative abundance of *Frankia* bacteria on roots (*n* = 25), **(e)** the diversity of N cycling genes in soil as explained by the relative abundance of *Frankia* bacteria on roots (*n* = 25), **(f)** the diversity of bacterial functional genes on roots as explained by the relative abundance of *Frankia* bacteria on roots (*n* = 25), **(g)** the relative abundance (normalized gene counts from metagenomic reads) of N fixing genes on roots as explained by the relative abundance of *Frankia* bacteria on roots (*n* = 25), **(h)** the ratio of bacteria/fungi (metagenomic reads) in soil as explained by the relative abundance of *Frankia* bacteria on roots (*n* = 25). Colors represent different tree hosts: *Betula pendula* (blue), *Sorbus* aucuparia (red), *S. torminalis* (orange), *S. domestica* (yellow), *Alnus glutinosa* (light green), and *A. incana* (dark green). Data points (*n*) presented in **a-c** are mean values of individual tree samples (> 10 m apart) from biologically independent tree species at each independent site, and error bars represent +/- the standard

error of the mean. Data points (*n*) presented in **d-h** are composite values of pooled individual tree samples (> 10 m apart) from biologically independent tree species at each independent site (i.e., *n* = tree species X site). The marginal R^2 (R^2m) of the fixed effect and *p* values (calculated using the Satterthwaite approximation in a two-sample *t*-test) from linear mixed-effects models (with plot embedded in site crossed with tree species as random effects) are listed, and the standard error of the fitted line is shaded grey. The statistical test used was two-sided. For linear-mixed effects model summaries supporting this figure see Supplementary Data 14.

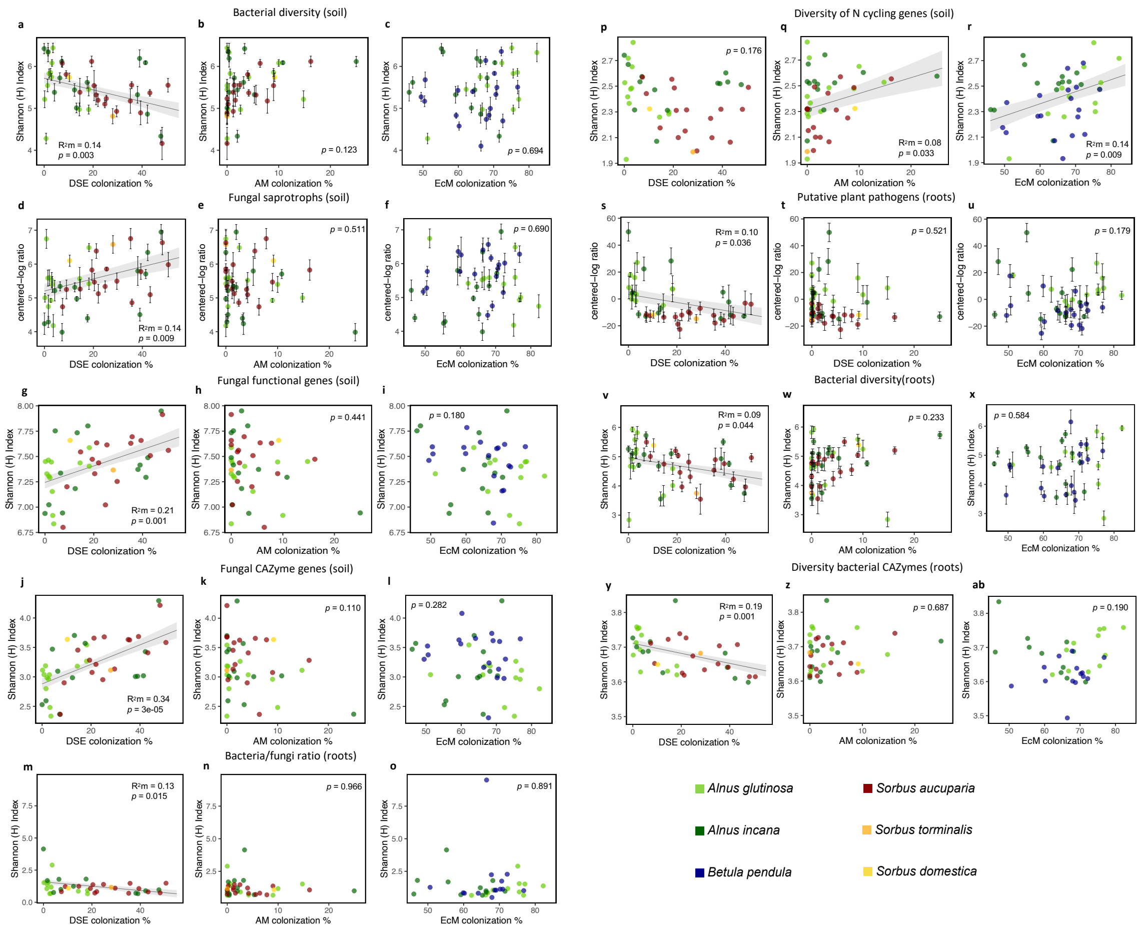

Fig. S3 Results of linear mixed-effects models showing bacterial diversity (Shannon H Index) in soil as explained by **(a)** dark sepatate endophyte (DSE) colonization (*n* = 43), **(b)** arbuscular mycorrhizal (AM) colonization (*n* = 43), and **(c)** ectomycorrhizal (EcM) colonization (*n* = 43); the relative abundance (centered-log ratio of metabarcoding reads) of soil fungal saprotrophs as explained by **(d)** DSE colonization (*n* = 43), **(e)** AM colonization (*n* = 43), and **(f)** EcM colonization (*n* = 43); the diversity of total fungal functional genes in soil as explained by **(g)** DSE colonization (*n* = 43), **(h)** AM colonization (*n* = 43), and **(i)** EcM colonization (*n* = 43); the diversity of fungal carbohydrate-active enzymes (CAZymes) in soil as explained by **(j)** DSE colonization (*n* = 43), **(k)** AM colonization (*n* = 43), and **(l)** EcM colonization (*n* = 43); the ratio of bacteria/fungi (metagenomic reads) in roots as explained by **(m)** DSE colonization (*n* = 39), **(n)** AM colonization (*n* = 39), and **(o)** EcM colonization (*n* = 36); the diversity of nitrogen (N) cycling genes in soil as explained by **(p)**

DSE colonization (n = 43), **(q)** AM colonization (n = 43), and **(r)** EcM colonization (n = 43); the relative abundance of putative fungal plant pathogens on roots as explained by **(s)** DSE colonization (n = 43), **(t)** AM colonization (n = 43), and **(u)** EcM colonization (n = 43); bacterial diversity on roots as explained by **(v)** DSE colonization (n = 43), **(w)** AM colonization (n = 43), and **(x)** EcM colonization (n = 43); and diversity of bacterial CAZymes in roots as explained by **(y)** DSE colonization (n = 39), **(z)** AM colonization (n = 39), and **(ab)** EcM colonization (n = 36). Colors represent different tree hosts: *Betula pendula* (blue), *Sorbus* aucuparia (red), *S. torminalis* (orange), *S. domestica* (yellow), *Alnus glutinosa* (light green), and *A. incana* (dark green). Data points (n) presented in **a-f** and **s-x** are mean values of individual tree samples (> 10 m apart) from biologically independent tree species at each independent site, and error bars represent +/- the standard error of the mean. Data points (n) presented in **g-r** and **y-ab** are composite values of pooled individual tree samples (> 10 m apart) from biologically independent tree species at each independent site (i.e., n = tree species X site). The marginal R^2 (R^2m) of the fixed effect and p values (calculated using the Satterthwaite approximation in a two-sample t-test) from linear mixed-effects models (with plot embedded in site crossed with tree species as random effects) are listed, and the standard error of the fitted line is shaded grey. The statistical test used was two-sided. For linear-mixed effects model summaries supporting this figure see Supplementary Data 15.

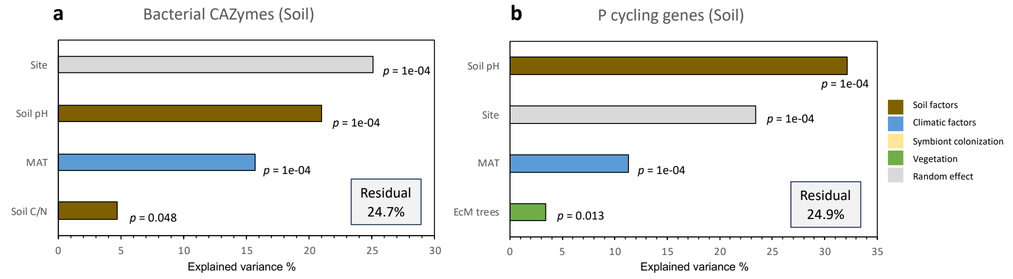

Fig. S4 Results of permutational multivariate analysis of variance (PERMANOVA) (9999 permutations) on factors explaining the composition of **(a)** bacterial CAZymes in soil (n = 61) and **(b)** P cycling genes in soil (n = 61). Bray-Curtis's dissimilarity was used for normalized gene count tables. Soil factors (brown) are soil pH, soil C/N, and soil moisture; climatic factors (blue) are MAT, MAP, and CMD; root symbiont colonization factors (yellow) are DSE colonization, EcM colonization, and AM colonization; vegetation factors (green) are the basal area of EcM/AM trees, and the basal area of coniferous EcM trees. Variance explained by site and tree species as random effects is shaded grey, and residual variance is listed in the grey boxes. Only individual factors that were significant ($p < 0.05$) are displayed and their p-values (999 permutations) are listed adjacent the factor, for detailed PERMANOVA results see Supplementary Data 4. The statistical test used was two-sided.

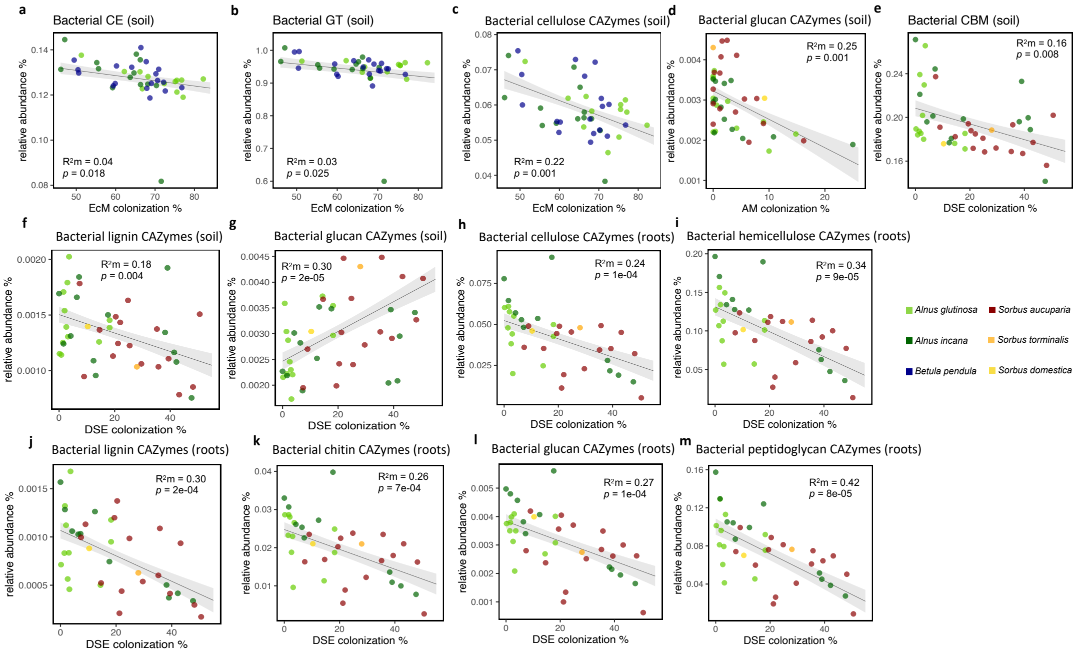

Fig. S5 Results of linear mixed-effects models showing **(a)** the relative abundance (% of total metagenomic reads) of bacterial carbohydrate esterases (CE) in soil as explained by ectomycorrhizal (EcM) colonization (*n* = 43), **(b)** the relative abundance of bacterial glycosyltransferases (GT) in soil as explained by EcM colonization (*n* = 43), **(c)** the relative abundance of bacterial CAZymes targeting cellulose in soil as explained by EcM colonization (*n* = 43), **(d)** the relative abundance of bacterial CAZymes targeting glucans in soil as explained by arbuscular mycorrhizal (AM) colonization (*n* = 43), **(e)** the relative abundance of bacterial carbohydrate-binding modules (CBM) in soil as explained by dark septate endophyte (DSE) colonization (*n* = 43), **(f)** the relative abundance of bacterial carbohydrate active enzymes (CAZymes) targeting lignin in soil as explained by DSE colonization (*n* = 43), **(g)** the relative abundance of bacterial CAZymes targeting glucans in soil as explained by DSE colonization (*n* = 43), **(h)** the relative abundance of bacterial CAZymes targeting cellulose in roots as explained by DSE colonization (*n* = 39), **(i)** the relative abundance of bacterial CAZymes targeting hemicellulose in roots as explained by DSE colonization (*n* = 39), **(j)** the relative abundance of bacterial CAZymes targeting lignin in roots as explained by DSE colonization (*n* = 39), **(k)** the relative abundance of bacterial CAZymes targeting chitin

in roots as explained by DSE colonization (*n* = 39), **(l)** the relative abundance of bacterial CAZymes targeting glucans in roots as explained by DSE colonization (*n* = 39), and **(m)** the relative abundance of bacterial CAZymes targeting peptidoglycans in roots as explained by DSE colonization (*n* = 39). Colors represent different tree hosts: *Betula pendula* (blue), *Sorbus* aucuparia (red), *S. torminalis* (orange), *S. domestica* (yellow), *Alnus glutinosa* (light green), and *A. incana* (dark green). Data points (*n*) presented are composite values of pooled individual tree samples (> 10 m apart) from biologically independent tree species at each independent site (i.e., *n* = tree species X site). The marginal R^2 (R^2m) of the fixed effect and *p* values (calculated using the Satterthwaite approximation in a two-sample *t*-test) from linear mixed-effects models (with plot embedded in site crossed with tree species as random effects) are listed, and the standard error of the fitted line is shaded grey. The statistical test used was two-sided. The statistical test used was two-sided. For linear-mixed effects model summaries supporting this figure see Supplementary Data 16.

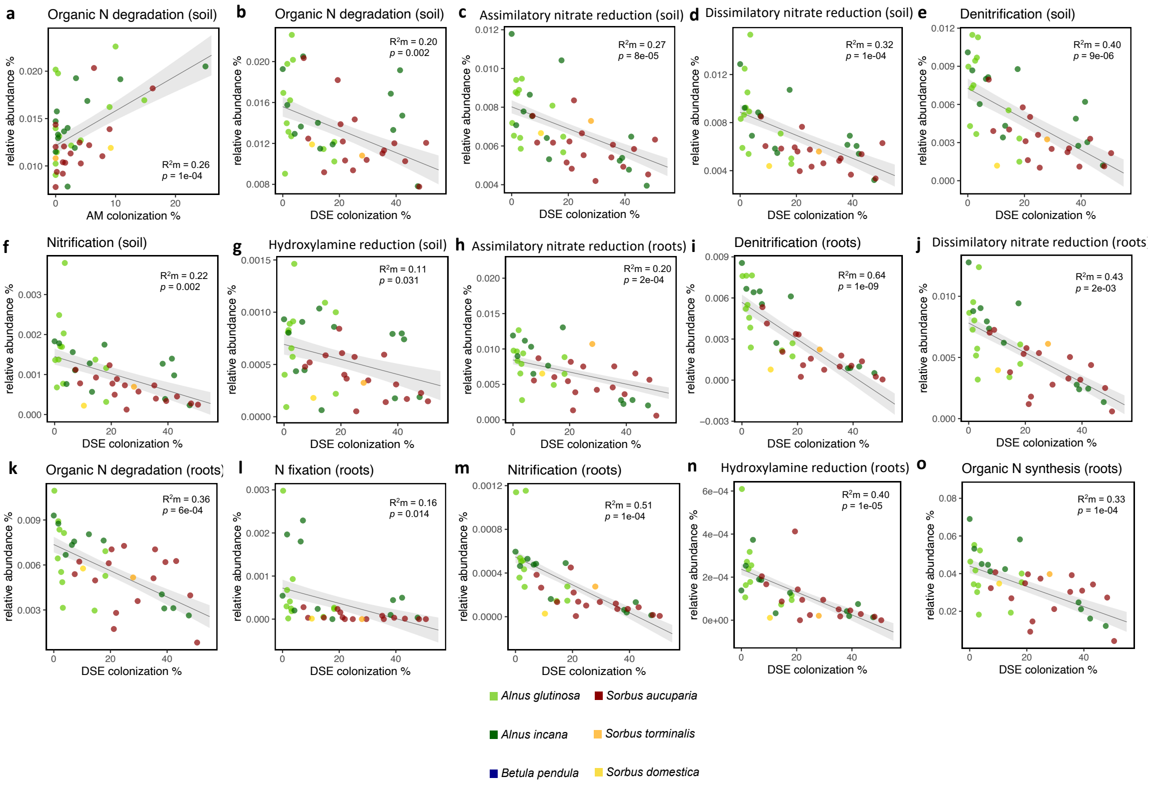

Fig. S6 Results of linear mixed-effects models showing **(a)** the relative abundance (% of total metagenomic reads) of organic nitrogen (N) degradation genes in soil as explained by AM colonization (n = 43), **(b)** the relative abundance of organic N degradation genes in soil as explained by dark septate endophyte (DSE) colonization (n = 43), **(c)** the relative abundance of assimilatory nitrate reduction genes in soil as explained by DSE colonization (n = 43), **(d)** the relative abundance of dissimilatory nitrate reduction genes in soil as explained by DSE colonization (n = 43), **(e)** the relative abundance of denitrification genes in soil as explained by DSE colonization (n = 43), **(f)** the relative abundance of nitrification genes in soil as explained by DSE colonization (n = 43), **(g)** the relative abundance of hydroxylamine reduction genes in soil as explained by DSE colonization (n = 43), **(h)** the relative abundance of assimilatory nitrate reduction genes in roots as explained by DSE colonization (n = 39), **(i)** the relative abundance of denitrification genes in roots as explained by DSE colonization (n = 39), **(j)** the relative abundance of dissimilatory nitrate reduction in roots as explained by DSE

colonization (n = 39), **(k)** the relative abundance of organic N degradation genes in roots as explained by DSE colonization (n = 39), **(l)** the relative abundance of N fixation genes in roots as explained by DSE colonization (n = 39), **(m)** the relative abundance of nitrification genes in roots as explained by DSE colonization (n = 39), **(n)** the relative abundance of hydroxylamine reduction genes in roots as explained by DSE colonization (n = 39), and **(o)** the relative abundance of organic N synthesis genes in roots as explained by DSE colonization (n = 39). Colors represent different tree hosts: *Betula pendula* (blue), *Sorbus* aucuparia (red), *S. torminalis* (orange), *S. domestica* (yellow), *Alnus glutinosa* (light green), and *A. incana* (dark green). Data points (n) presented are composite values of pooled individual tree samples (> 10 m apart) from biologically independent tree species at each independent site (i.e., n = tree species X site). The marginal R^2 (R^2m) of the fixed effect and p values (calculated using the Satterthwaite approximation in a two-sample t-test) from linear mixed-effects models (with plot embedded in site crossed with tree species as random effects) are listed, and the standard error of the fitted line is shaded grey. The statistical test used was two-sided. For linear-mixed effects model summaries supporting this figure see Supplementary Data 17.

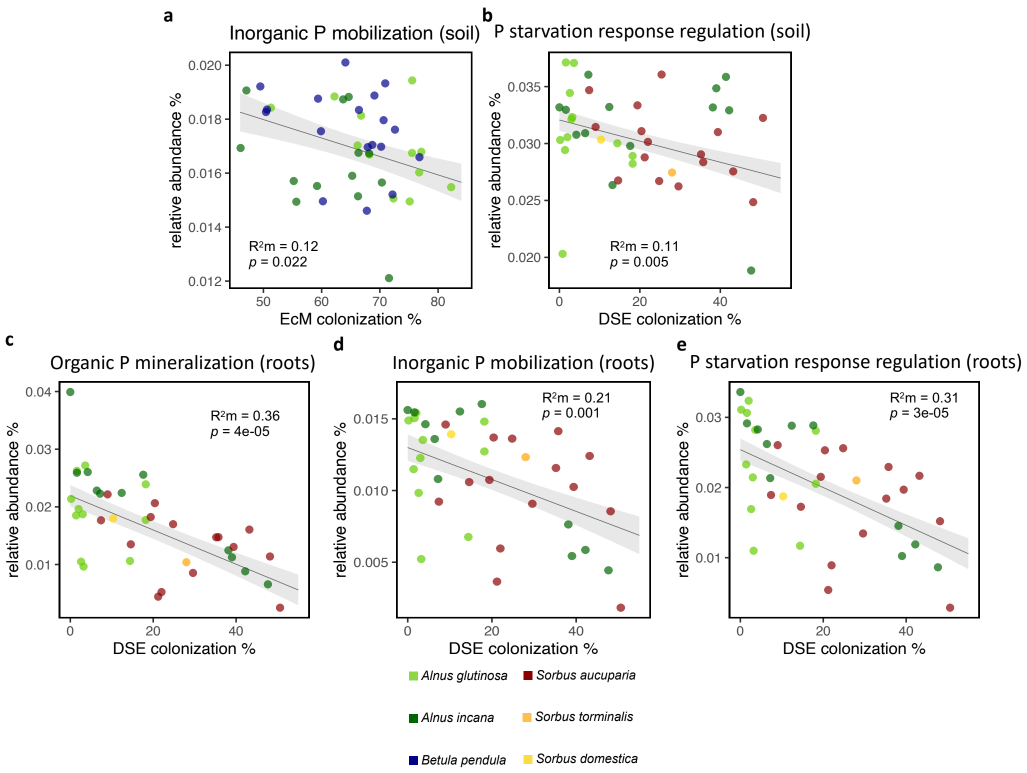

Fig. S7 Results of linear mixed-effects models showing **(a)** the relative abundance (% of total metagenomic reads) of inorganic phosphorus (P) mobilization genes in soil as explained by ectomycorrhizal (EcM) colonization (*n* = 43), **(b)** the relative abundance of P starvation response regulation genes in soil as explained by dark septate endophyte (DSE) colonization (*n* = 43), **(c)** the relative abundance of organic P mineralization genes in roots as explained by DSE colonization (*n* = 39), **(d)** the relative abundance of inorganic P mobilization genes in roots as explained by DSE colonization (*n* = 39), and **(e)** the relative abundance of P starvation response regulation genes in roots as explained by DSE colonization (*n* = 39). Colors represent different tree hosts: *Betula pendula* (blue), *Sorbus* aucuparia (red), *S. torminalis* (orange), *S. domestica* (yellow), *Alnus glutinosa* (light green), and *A. incana* (dark green). Data points (*n*) presented are composite values of pooled individual tree samples (>

10 m apart) from biologically independent tree species at each independent site (i.e., n = tree species X site). The marginal R^2 (R^2m) of the fixed effect and p values (calculated using the Satterthwaite approximation in a two-sample t-test) from linear mixed-effects models (with plot embedded in site crossed with tree species as random effects) are listed, and the standard error of the fitted line is shaded grey. The statistical test used was two-sided. For linear-mixed effects model summaries supporting this figure see Supplementary Data 18.

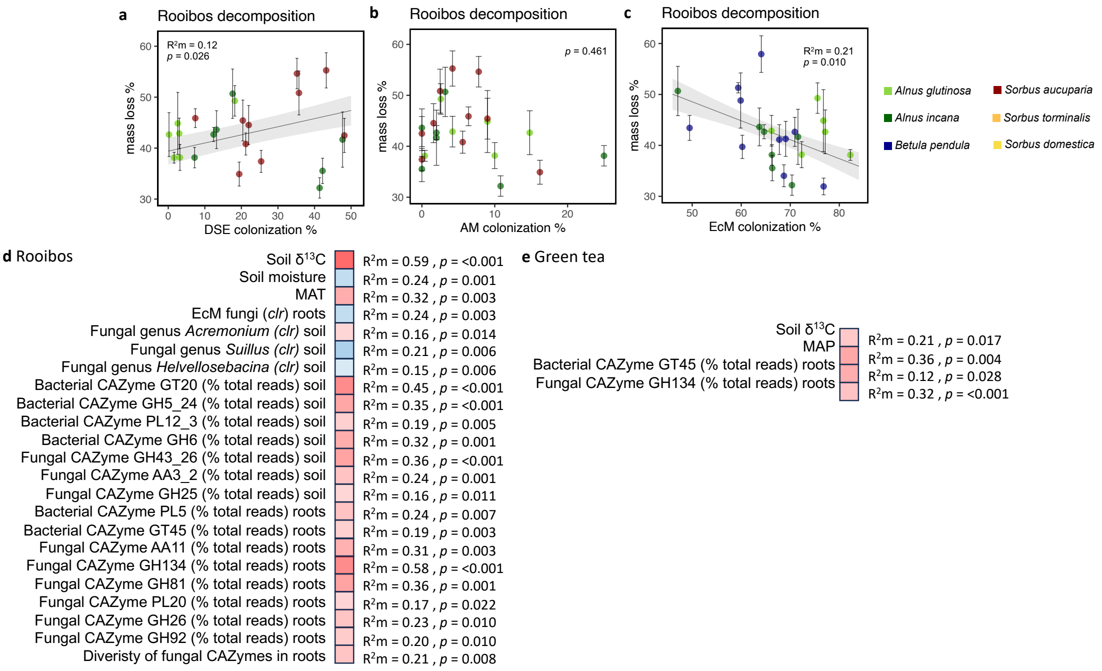

Fig. S8 Results of linear mixed-effects models showing **(a)** the mass loss (%) of rooibos teabags as explained by dark septate endophyte (DSE) colonization (*n* = 23), **(b)** the mass loss (%) of rooibos teabags as explained by arbuscular mycorrhizal (AM) colonization (*n* = 23), **(c)** the mass loss (%) of rooibos teabags as explained by ectomycorrhizal (EcM) colonization (*n* = 23), **(d)** the mass loss (%) of rooibos teabags as explained by the best set of climate, soil and biotic predictors, and **(c)** the mass loss (%) of green tea teabags as explained by the best set of climate, soil and biotic predictors. Colors represent different tree hosts: *Betula pendula* (blue), *Sorbus* aucuparia (red), *S. torminalis* (orange), *S. domestica* (yellow), *Alnus glutinosa* (light green), and *A. incana* (dark green). Data points (*n*) presented are mean values of individual tree samples (> 10 m apart) from biologically independent tree species at each independent site, and error bars represent +/- the standard error of the mean. The marginal R^2 (R^2m) of the fixed effect and *p* values (calculated using the Satterthwaite approximation in a two-sample *t*-test) from linear mixed-effects models (with plot embedded in site crossed with tree species as random effects) are listed, and the standard error of the

fitted line is shaded grey. Red colored squares indicate a positive relationship and blue colored squares indicate a negative relationship; the saturation indicates the strength of the relationship. The statistical test used was two-sided. For linear-mixed effects model summaries supporting this figure see Supplementary Data 19.

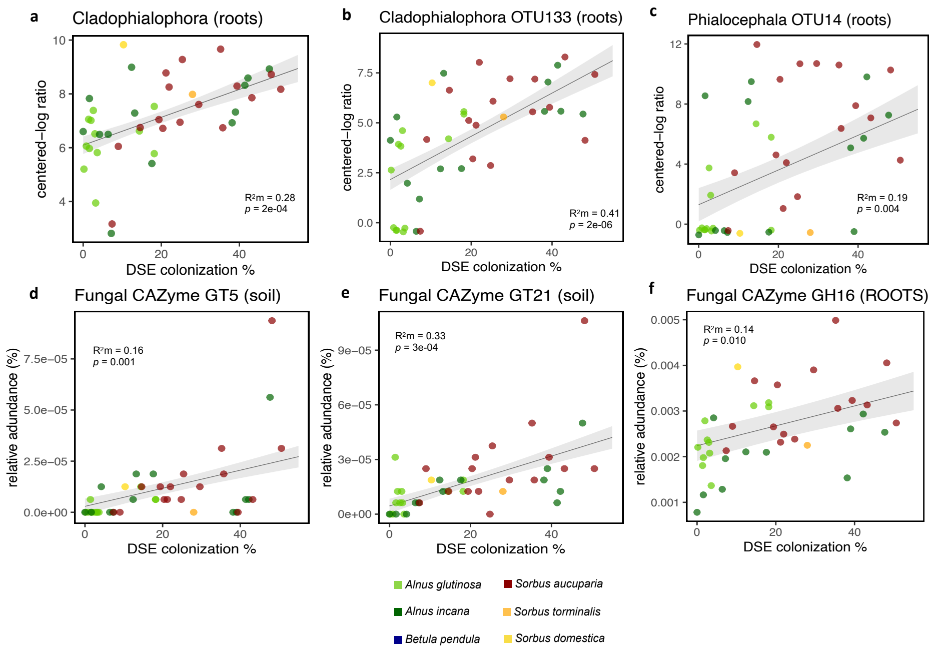

Fig. S9 Results of linear mixed-effects models showing **(a)** the relative abundance (centered-log ratio of metabarcoding reads) of the fungal genus *Cladophialophora* in roots as explained by dark septate endophyte (DSE) colonization (n = 43), **(b)** the relative abundance (centered-log ratio of metabarcoding reads) of the fungal OTU *Cladophialophora* OTU133 in roots as explained by DSE colonization (n = 43), **(c)** the relative abundance (centered-log ratio of metabarcoding reads) of the fungal OTU *Phialocephala* OTU14 in roots as explained by DSE colonization (n = 43), **(d)** the relative abundance (% of total metagenomic reads) of the fungal carbohydrate active enzyme (CAZyme) family GT5 in soil as explained by DSE colonization (n = 43), **(e)** the relative abundance (% of total metagenomic reads) of the fungal CAZyme family GT21 in soil as explained by DSE colonization (n = 43), and **(f)** the relative abundance (% of total reads) of the fungal CAZyme family GH16 in roots as explained by DSE colonization (n = 39). Colors represent different tree hosts: *Betula pendula* (blue), *Sorbus* aucuparia (red), *S. torminalis* (orange), *S. domestica* (yellow), *Alnus glutinosa* (light green), and *A. incana* (dark green). Data points (n) presented are composite values of pooled individual tree samples (> 10 m apart) from biologically independent tree species at each

independent site (i.e., *n* = tree species X site). The marginal R^2 (R^2m) of the fixed effect and *p* values (calculated using the Satterthwaite approximation in a two-sample *t*-test) from linear mixed-effects models (with plot embedded in site crossed with tree species as random effects) are listed, and the standard error of the fitted line is shaded grey. The statistical test used was two-sided. For linear-mixed effects model summaries supporting this figure see Supplementary Data 20.

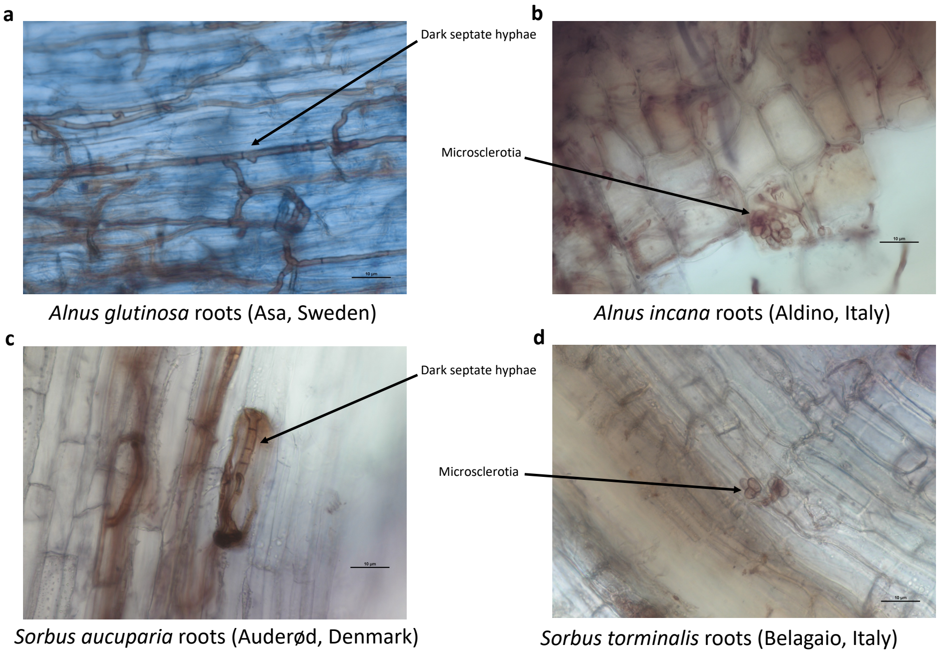

Fig. S10 Photographs of dark septate endophyte structures in roots in this study stained with trypan blue in an acidic glycerol solution analyzed under X 400 magnification: **(a)** *Alnus glutinosa* roots with colonized by dark septate hyphae, **(b)** *Alnus incana* roots with an example of dark septate endophyte microsclerotia, **(c)** *Sorbus aucuparia* roots colonized by dark septate hyphae, **(d)** *Sorbus torminalis* roots with an example of dark septate endophyte microsclerotia. Scale of the images is indicated by the black scale bars (10 µm). All photos were taken by Jane Oja.

Supplementary references

1. Soudzilovskaia, N.A., et al., *Global patterns of plant root colonization intensity by mycorrhizal fungi explained by climate and soil chemistry.* Global Ecology and Biogeography, 2015. **24**(3): p. 371-382.
2. Lansac, A., A. Martin, and A. Roldan, *Mycorrhizal colonization and drought interactions of Mediterranean shrubs under greenhouse conditions.* Arid Land Research and Management, 1995. **9**(2): p. 167-175.
3. Kennedy, P.G. and K.G. Peay, *Different soil moisture conditions change the outcome of the ectomycorrhizal symbiosis between Rhizopogon species and Pinus muricata.* Plant and Soil, 2007. **291**(1): p. 155-165.
4. Kilpeläinen, J., et al., *Does severe soil drought have after-effects on arbuscular and ectomycorrhizal root colonisation and plant nutrition?* Plant and Soil, 2017. **418**(1): p. 377-386.
5. Tedersoo, L. and M. Bahram, *Mycorrhizal types differ in ecophysiology and alter plant nutrition and soil processes.* Biological Reviews, 2019. **94**(5): p. 1857-1880.
6. di Pietro, M., J.-L. Churin, and J. Garbaye, *Differential ability of ectomycorrhizas to survive drying.* Mycorrhiza, 2007. **17**(6): p. 547-550.
7. García de Jalón, L., et al., *Microhabitat and ectomycorrhizal effects on the establishment, growth and survival of Quercus ilex L. seedlings under drought.* PLOS ONE, 2020. **15**(6): p. e0229807.
8. Finlay, R.D., *Ecological aspects of mycorrhizal symbiosis: with special emphasis on the functional diversity of interactions involving the extraradical mycelium.* Journal of Experimental Botany, 2008. **59**(5): p. 1115-1126.
9. Han, L., et al., *Plant identity and soil variables shift the colonisation and species composition of dark septate endophytes associated with medicinal plants in a northern farmland in China.* Applied Soil Ecology, 2021. **167**: p. 104042.
10. Pandey, A., *Are dark septate endophytes bioindicators of climate in mountain ecosystems?* Rhizosphere, 2019. **9**: p. 110-111.
