# Supplementary material for: Pervasive associations between dark septate endophytic fungi with tree root and soil microbiomes across Europe
Source: Nat Commun. 2024 Jan 2;15:159. doi: 10.1038/s41467-023-44172-4 (PMC10761831; doi:10.1038/s41467-023-44172-4)
Supplement: Supplementary file 3 — Description of Additional Supplementary Files [file 41467_2023_44172_MOESM3_ESM.pdf]

Description of Additional Supplementary Files

File Name: Supplementary Data 1
Description: Information about the locations, sites, plots, and tree species used in this study.

File Name: Supplementary Data 2
Description: Fungal operational taxonomic units (OTUs) assigned as potential dark septet endophyte (DSE) taxa in this study from roots and soil, including their ITS2 sequences and taxonomic information.

File Name: Supplementary Data 3
Description: Detailed summary output of piecewise structural equation modeling (SEM) performed in this study and displayed in Fig. 4. The statistical test used was two-sided.

File Name: Supplementary Data 4
Description: Detailed summary output of permutational analysis of variance (PERMANOVA) performed in this study and displayed in Fig. 5 and Fig. S4. The statistical test used was two-sided.

File Name: Supplementary Data 5
Description: Spearman rank correlation analysis results for general functional genes (COG and EGGNOG) from roots and soil and their correlations with dark septate endophyte (DSE) colonization. Only correlations with a p-value less than 0.005 are listed to account for multiple comparisons. The statistical test used was two-sided.

File Name: Supplementary Data 6
Description: Total metagenomic reads per sample from root and soil metagenome samples used in this study

File Name: Supplementary Data 7
Description: Carbohydrate-active enzymes analyzed in this study according to biomass source and substrate

File Name: Supplementary Data 8
Description: Nitrogen cycling genes analyzed in this study and grouped according to process

File Name: Supplementary Data 9
Description: Phosphorus cycling genes analyzed in this study and grouped according to process

File Name: Supplementary Data 10
Description: Fig. 2 summary results of linear mixed-effects models

File Name: Supplementary Data 11
Description: Fig. 3 summary results of linear mixed-effects models

File Name: Supplementary Data 12
Description: Fig. 6 summary results of linear models

File Name: Supplementary Data 13
Description: Fig. S1 summary results of linear mixed-effects models

File Name: Supplementary Data 14
Description: Fig. S2 summary results of linear mixed-effects models

File Name: Supplementary Data 15
Description: Fig. S3 summary results of linear mixed-effects models

File Name: Supplementary Data 16
Description: Fig. S5 summary results of linear mixed-effects models

File Name: Supplementary Data 17
Description: Fig. S6 summary results of linear mixed-effects models

File Name: Supplementary Data 18
Description: Fig. S7 summary results of linear mixed-effects models

File Name: Supplementary Data 19
Description: Fig. S8 summary results of linear mixed-effects models

File Name: Supplementary Data 20
Description: Fig. S9 summary results of linear mixed-effects models
