# Supplementary material for: Pervasive associations between dark septate endophytic fungi with tree root and soil microbiomes across Europe
Source: Nat Commun. 2024 Jan 2;15:159. doi: 10.1038/s41467-023-44172-4 (PMC10761831; doi:10.1038/s41467-023-44172-4)
Supplement: Supplementary file 24 — Reporting Summary [file 41467_2023_44172_MOESM24_ESM.pdf]

nature portfolio

Corresponding author(s): Tarquin Netherway

Last updated by author(s): Nov 26, 2023

Reporting Summary

Nature Portfolio wishes to improve the reproducibility of the work that we publish. This form provides structure for consistency and transparency in reporting. For further information on Nature Portfolio policies, see our Editorial Policies and the Editorial Policy Checklist.

Statistics

For all statistical analyses, confirm that the following items are present in the figure legend, table legend, main text, or Methods section.

n/a	Confirmed	
☐	☒	The exact sample size (*n*) for each experimental group/condition, given as a discrete number and unit of measurement
☐	☒	A statement on whether measurements were taken from distinct samples or whether the same sample was measured repeatedly
☐	☒	The statistical test(s) used AND whether they are one- or two-sided<br>*Only common tests should be described solely by name; describe more complex techniques in the Methods section.*
☐	☒	A description of all covariates tested
☐	☒	A description of any assumptions or corrections, such as tests of normality and adjustment for multiple comparisons
☐	☒	A full description of the statistical parameters including central tendency (e.g. means) or other basic estimates (e.g. regression coefficient) AND variation (e.g. standard deviation) or associated estimates of uncertainty (e.g. confidence intervals)
☐	☒	For null hypothesis testing, the test statistic (e.g. *F*, *t*, *r*) with confidence intervals, effect sizes, degrees of freedom and *P* value noted<br>*Give P values as exact values whenever suitable.*
☒	☐	For Bayesian analysis, information on the choice of priors and Markov chain Monte Carlo settings
☐	☒	For hierarchical and complex designs, identification of the appropriate level for tests and full reporting of outcomes
☐	☒	Estimates of effect sizes (e.g. Cohen's *d*, Pearson's *r*), indicating how they were calculated

Our web collection on statistics for biologists contains articles on many of the points above.

Software and code

Policy information about availability of computer code

Data collection	The pipeline to process metabarcoding samples is available under https://lotus2.earlham.ac.uk/main.php?site=downloads (https://github.com/hildebra/lotus2). The pipeline to process shotgun metagenomic samples is available under https://github.com/hildebra/MATAFILER (https://doi.org/10.5281/zenodo.5831723). The carbohydrate active enzymes (CAZy) Database is available under http://www.cazy.org/, the nitrogen cycling gene (NCyc) Database is available under https://github.com/qichao1984/NCyc (https:doi.org/10.1093/bioinformatics/bty741), the phosphorus cycling gene (PCyCDB) Database is available under https://github.com/ZengJiaxiong/Phosphorus-cycling-database<br>(https://doi.org/10.1186/s40168-022-01292-1). The Evolutionary genealogy of genes: Non-supervised Orthologous Groups (EggNOG) Database is available under http://eggnog5.embl.de/#/app/home. The Kyoto Encyclopedia of Genes and Genomes (KEGG) Database is available under https://www.genome.jp/kegg/kegg1.html.
Data analysis	For data analysis we used RStudio version 1.4.1717 and Primer V7 with PERMANOVA+

For manuscripts utilizing custom algorithms or software that are central to the research but not yet described in published literature, software must be made available to editors and reviewers. We strongly encourage code deposition in a community repository (e.g. GitHub). See the Nature Portfolio guidelines for submitting code & software for further information.

Data

Policy information about availability of data

All manuscripts must include a data availability statement. This statement should provide the following information, where applicable:

- Accession codes, unique identifiers, or web links for publicly available datasets
- A description of any restrictions on data availability
- For clinical datasets or third party data, please ensure that the statement adheres to our policy

The 16S and ITS metabarcoding data generated in this study have been deposited in the NCBI Sequence Read Archive (SRA) under the Bio Project accession number: PRJNA1021963 [https://www.ncbi.nlm.nih.gov/bioproject/PRJNA1021963]. All root and soil metagenomic sequences and associated metadata have been deposited in the European Nucleotide Archive (ENA) under accession number: PRJEB56450 [https://www.ebi.ac.uk/ena/browser/view/PRJEB56450].
The carbohydrate active enzymes (CAZy) Database is available under http://www.cazy.org/, the nitrogen cycling gene (NCyc) Database is available under https://github.com/qichao1984/NCyc [https:doi.org/10.1093/bioinformatics/bty741], the phosphorus cycling gene (PCyCDB) Database is available under https://github.com/ZengJiaxiong/Phosphorus-cycling-database
[https://doi.org/10.1186/s40168-022-01292-1]. The Evolutionary genealogy of genes: Non-supervised Orthologous Groups (EggNOG) Database is available under http://eggnog5.embl.de/#/app/home. The Kyoto Encyclopedia of Genes and Genomes (KEGG) Database is available under https://www.genome.jp/kegg/kegg1.html. The data supporting the results and figures in this study are provided in the Supplementary Information/Source Data file. The FungalTraits tool used for fungal guild assignments is available under https://docs.google.com/spreadsheets/d/1cxImJWMYVTr6uIQXcTLwK1YNNzQvKJJifzzNpKCM6O0/edit?usp=sharing [https://doi.org/10.1007/s13225-020-00466-2]. The raw metabarcoding OTU tables with taxonomic information, functional gene matrices (metagenomics), root symbiont colonization rates, tea bag mass loss, and environmental metadata generated in this study are deposited in the Zenodo open data repository (CERN) under https://doi.org/10.5281/zenodo.10203817.

Research involving human participants, their data, or biological material

Policy information about studies with human participants or human data. See also policy information about sex, gender (identity/presentation), and sexual orientation and race, ethnicity and racism.

Reporting on sex and gender	N/A
Reporting on race, ethnicity, or other socially relevant groupings	N/A
Population characteristics	N/A
Recruitment	N/A
Ethics oversight	N/A

Note that full information on the approval of the study protocol must also be provided in the manuscript.

Field-specific reporting

Please select the one below that is the best fit for your research. If you are not sure, read the appropriate sections before making your selection.

☐ Life sciences ☐ Behavioural & social sciences ☒ Ecological, evolutionary & environmental sciences

For a reference copy of the document with all sections, see nature.com/documents/nr-reporting-summary-flat.pdf

Ecological, evolutionary & environmental sciences study design

All studies must disclose on these points even when the disclosure is negative.

Study description

This study utilizes high-throughput sequencing methods for metabarcoding and shotgun metagenomics of soil and root bacterial and fungal communities and their functional genes, soil physiochemical measurements, and tea bag litter decomposition analysis to look at the effects of different tree-fungal symbioses together with climate and soil on the structure and potential functions of the soil and root microbiomes underneath widespread tree species across a European latitudinal gradient. The study was conducted across a 3220 km latitudinal gradient, which included 18 locations that were either established research stations or vegetation reserves from northern Norway (69.46°N, 30.02°E) to central Italy (42.08°N, 9.82°E). Selected sites contained widespread deciduous broadleaf tree species from three genera (Alnus, Betula, and Sorbus), differing in their preference for different mycorrhizal associations and N-fixing status. Alnus incana was present at 13 sites from northern Norway to northern Italy; A. glutinosa at 12 sites from central Sweden to central Italy; Betula pendula was present at all sites; and Sorbus aucuparia was present at all but the two southernmost sites in Italy, where it was replaced by S. torminalis or S. domestica. At each site, 5 trees of each target species were selected with a diameter at breast height (DBH) of 10 and 20 cm, and a distance of at least 10 m within a 50 m radius of one another. If different target species were growing separately (> 50 m) or in clearly different habitats, we considered these habitats as separate plots, resulting in 11 sites with 1 plot, 3 sites with 2 plots, 3 sites with 3 plots, and 1 site with 4 plots, leading to a total of 30 plots across the 18 locations. There

	were no treatment factors or interactions considered in this study. The study has a hierarchical design where tree individuals are embedded in tree species per plot/site (total tree individuals across the study n = 305). No experiments were performed.
Research sample	Each research sample was a composite of 4 top soil samples (5 x 10 cm), or fine root samples < 2mm, taken underneath each target sampling tree at each site, and 1 rooibos teabag and 1 green tea bag (Lipton) left for one year underneath each sampled tree and retrieved after one year for mass loss analysis. In each soil and root sample we isolated DNA to amplify bacterial and fungal communities using PCR and amplicon sequencing and merged DNA samples from individual tree replicates per tree species per site to perform shotgun metagenomic sequencing on composite samples per tree species per site. Each target sampling tree was selected based on having a diameter at breast height (DBH) between 10 and 20 cm, and a distance of at least 10 m between other sampling trees within a 50 m radius of one another. 4 composite soil and root samples per sampling trees were merged to better capture the representative bacterial and fungal communities living under each tree, i.e., each sample aimed to capture the population of soil and root associated bacteria and fungi living underneath the selected individual trees and tree species across Europe.
Sampling strategy	We selected 18 sampling locations across Europe based on the presence of our target tree species, accessibility and sampling permissions , sampling was undertaken between the months of August and September 2019 primarily by the first author, with assistance from others. For sampling of soils and roots, and for putting down tea bags, five biologically independent replicate trees of each target species were selected at each site when present, tree individuals were deemed independent when they were growing more than 10 meters apart from one another, no statistical test was used to determine sampling size, instead we determined this based on the minimum number of target trees that were between 10-20 cm diameter present per tree species per at each site that were 10 meters apart , and we also considered the minimum feasible sample size for statistical analysis based on prior experience of the authors e.g. Polme et al. 2014 https://doi.org/10.1111/nph.12962. At 10 sites from Norway to Germany, one Lipton teabag with rooibos (C/N = 43) and one with green tea (C/N = 12) were buried to 10 cm deep at one soil sampling point per tree and collected after 12 months according to a modified version of the Teabag Index protocol [90]; a total of 284 out of 330 teabags were recovered (leaving at least three replicates per species per site) and dried at 70°C until constant weight to measure mass loss.
Data collection	Data was collected from soil and root samples, and tea bags mentioned above, molecular work was undertaken by the first author and additionally the UMBLA lab at SLU Uppsala, amplicon and shotgun metagenomic sequencing was performed on Illumina NovaSeq 6000 using 250 bp paired end reads by a commercial lab, tea bag decomposition was measured by the first author, soil nutrients were measured at a commercial lab, soil C,N and isotope analysis was performed by the third and sixth authors, analysis of root colonization by ectomycorrhizal fungi was performed by the first author, and root colonization analysis of arbuscular mycorrhizal and dark septate endophytic fungi was performed by the fifth author.
Timing and spatial scale	Soil and root sampling was undertaken once per site during a 28 day period from a 3900 km latitudinal gradient across western Europe between the 5th of August (N Norway) and the 11th of September 2019 (central Italy) from sites in a north south direction to roughly approximate a similar later stage in the growing season. Tea bags were collected at 12 months since 2019 sampling dates in 2020 according to a modified version of the Teabag Index protocol [90]; a total of 284 out of 330 teabags were recovered (leaving at least three replicates per species per site) and dried at 70°C until constant weight to measure mass loss.
Data exclusions	Samples from metabarcoding data that had less than 1000 reads were excluded
Reproducibility	Each site included several individual trees (n =5) of several target tree species (n = 3-4) per site (n =18), two successful technical PCR replicates were pooled per sample before metabarcoding sequencing. A total of 284 out of 330 teabags were recovered (leaving at least three replicates per species per site).
Randomization	We selected sites based on the presence of our target tree species, we selected individual trees based on their size (10-20 cm diameter) and distance from one another (at least 10 m apart) to avoid sampling the same soil microbial communities. Tree samples were allocated into experimental groups based on belonging to the same tree species and occuring at the same site, covariates were measured for each tree and accounted for in statistical analysis.
Blinding	Blinding was not performed as field sampling was undertaken in natural mixed forests were expertise was required to indentify tree species and to select and measure individual trees from which to sample.

Did the study involve field work? ☒ Yes ☐ No

Field work, collection and transport

Field conditions	Sampling was done under variable weather conditions which did not influence the work. Temperature and rainfall were obtained for the soil samples from the climate database and did not influence sampling time nor location. For detailed description, see Methods.
Location	Soil and root samples were taken from 18 locations across Europe including the countries of Norway, Finland, Sweden, Denmark, Germany and Italy see Fig 1, and for coordinates of sites see Supplementary Table 1
Access & import/export	Soil and roots samples were collected from locations in public domain or in previous agreement with the local community and/or property owner. The samples were exported from the origin countries and imported to Sweden, EU in cooperation with customs officers of the respective states, following the legal provisions of soil export and import, specifically exemptions for scientific purposes.
Disturbance	The field work caused minimal environmental disturbance associated with collecting small isolated amounts of soil and roots.

Reporting for specific materials, systems and methods

We require information from authors about some types of materials, experimental systems and methods used in many studies. Here, indicate whether each material, system or method listed is relevant to your study. If you are not sure if a list item applies to your research, read the appropriate section before selecting a response.

Materials & experimental systems

n/a	Involved in the study
☒	☐ Antibodies
☒	☐ Eukaryotic cell lines
☒	☐ Palaeontology and archaeology
☒	☐ Animals and other organisms
☒	☐ Clinical data
☒	☐ Dual use research of concern
☒	☐ Plants

Methods

n/a	Involved in the study
☒	☐ ChIP-seq
☒	☐ Flow cytometry
☒	☐ MRI-based neuroimaging

Plants

Seed stocks	N/A
Novel plant genotypes	N/A
Authentication	N/A
